# Efficacy of different strategies to treat anemia in children: a randomized clinical trial

**DOI:** 10.1186/1475-2891-9-40

**Published:** 2010-09-23

**Authors:** Jorge L Rosado, Karla E González, María del C Caamaño, Olga P García, Roxana Preciado, Mauricio Odio

**Affiliations:** 1 Facultad de Ciencias Naturales, Universidad Autónoma de Querétaro. Querétaro, México; 2 CINDETEC, Querétaro México; 3 Procter and Gamble, Cincinnati Ohio, USA

## Abstract

**Background:**

Anemia continues to be a major public health problem among children in many regions of the world, and it is still not clear which strategy to treat it is most effective.

**Objective:**

To evaluate the efficacy and children's acceptance of several recognized strategies to treat anemia.

**Methods:**

Non-breastfed children (n = 577), 6 to 43 mo of age, were screened for the trial; 267 were anemic (hemoglobin < 11.7 g/dL), and 266 of those were randomized into 1 of 5 treatments to received daily either: an iron supplement (IS), an iron+folic acid supplement (IFS), a multiple micronutrient supplement (MMS), a micronutrient-fortified complementary food as porridge powder (FCF), or zinc+iron+ascorbic acid fortified water (FW). The iron content of each daily dose was 20, 12.5, 10, 10 and 6.7 mg respectively. Hemoglobin (Hb), ferritin, total iron, weight and height were measured at baseline and after 4 months of treatment. Morbidity, treatment acceptability and adherence were recorded during the intervention.

**Results:**

All treatments significantly increased Hb and total iron concentration; ferritin did not change significantly. Groups MMS, IS and IFS increased Hb (g/dL) [1.50 (95%CI: 1.17, 1.83), 1.48 [(1.18, 1.78) and 1.57 (1.26, 1.88), respectively] and total iron ((μg/dL) [0.15 (0.01, 0.29), 0.19 (0.06, 0.31) and 0.12(-0.01, 0.25), respectively] significantly more than FCF [0.92 (0.64, 1.20)] but not to FW group [0.14 (0.04, 0.24)]. The prevalence of anemia was reduced to a greater extent in the MMS and IFS groups (72% and 69%, respectively) than in the FCF group (45%) (p < 0.05). There were no significant differences in anthropometry or in the number of episodes of diarrhea and respiratory infections among treatment groups. The supplements MMS and IS were less acceptable to children, than IFS, FCF and FW.

**Conclusion:**

The three supplements IS, ISF and MMS increased Hb more than the FCF; the supplements that contained micronutrients (IFS and MMS) were more effective for reducing the prevalence of anemia. In general, fortified foods were better accepted by the study participants than supplements.

**ClinicalTrial.gov Identifier:**

NCT00822380

## Background

Micronutrient deficiencies continue to be a major public health problem in many regions of the world. There have been an increasing number of epidemiological studies demonstrating the high prevalence of micronutrient deficiencies in different countries [1-3]. Other studies have focused on the functional and health consequences of micronutrient deficiencies [4,5]. However, more longitudinal studies are needed to address important issues on how to treat and prevent such deficiencies.

Among the consequences of micronutrient deficiencies in different populations around the world, anemia is perhaps the single best identified syndrome suffered by a high proportion of the population. Anemia is present in about 25% of the world's population [6]. It has been estimated that about 245 million children from 0 to 59 months of age are anemic in the world [7], and that approximately 50% of the anemia is attributable to iron deficiency [8,9]. Iron deficiency anemia (IDA) is present in developing and in developed countries as well [10]. It is known that other micronutrients deficiencies can contribute to anemia in addition to iron [11]. In a previous study in low income populations in Mexico, about 30% of anemic subjects did not respond to iron supplementation alone [12], suggesting that other nutrient deficiencies might also be involved.

Several alternatives are available for prevention and/or treatment of anemia. Iron supplementation with different iron sources, particularly ferrous sulfate, has been recommended and used in many regions of the world for several years [9,13]. More recently, the addition of other micronutrients to iron has been suggested. The United Nations Children's Fund (UNICEF) recommends the utilization of an iron and folic acid supplement [14]. The use of complementary foods as a safer form to deliver iron and other micronutrients has also been explored in different countries [15]. In Mexico, a milk-based powdered supplement that contains iron, zinc, copper and several vitamins was developed to treat and prevent micronutrient deficiencies and anemia in low-income populations [16,17]. Iron fortification of foods and beverages is another alternative to treat and prevent anemia and today it is a common practice to add micronutrients to different foods such as cereals, dairy foods, snacks and beverages [16,18-21].

It is believed that an iron supplement or an iron fortified food with added micronutrients will have a beneficial effect on hemoglobin (Hb) status in children at risk of micronutrient deficiencies. However, despite all the different strategies developed to treat and prevent anemia it is still not clear which strategy is more effective in children in terms of adherence and efficacy. The objective of the present study was to evaluate the efficacy and children's acceptance of several strategies that have recently been recommended to treat anemia.

## Methods

### Subjects and place of the study

The study was carried out in 4 rural communities within 50 kilometers of the city of Queretaro in Mexico: La Fuente, Los Cerritos, El Tejocote and Fuentezuelas. A census of all families in these communities was done before the study began; 2 months later, mothers of 577 children aged 6 to 42 mo were invited to participate. Study details and potential risks and benefits were explained to the mother or caretaker of each child, and they voluntarily signed informed consent forms allowing their children to participate. Hemoglobin concentration was measured in all children from a capillary blood sample after an overnight fast, and only those with anemia (Hb < 11.7 g/dL) were included in the efficacy study. Exclusion criteria also included exclusive breastfeeding or consumption of industrialized formula, chronic gastroenteritis or any other severe illness. Siblings from an anemic child were included in the study regardless of their anemia status but only anemic children were included in the statistical analyses. Parents of eligible children agreed not to give any other nutritional supplement to their children while they were enrolled in the study. Clinical evaluations and blood sample collections were done at the health clinics in the communities which depend on the Ministry of Health. The study protocol was reviewed and approved by the Bioethics Committee of the University of Queretaro.

A sample size of 53 children per treatment group was calculated to detect a difference of 0.6 g/dL in Hb change with a standard deviation of 1.0 g/dL, an alpha error of 0.05, a statistical power of 80% and a drop out rate of 20%.

### Experimental treatments and design

Anemic subjects were randomized into 1 of 5 treatment groups. Ten groups of children, who entered the study one at a time, were randomized independently to ensure treatment balance within each group. Randomization procedures also ensured homogeneous groups according to Hb status, age and gender. Siblings were allocated to the same treatment to facilitate treatment administration by the mother. The randomization process was done using a program specifically developed for the purpose of this study in SAS version 8.1 (SAS Institute Inc. Cary, NC) by personnel who were not in contact with the subjects or fieldworkers.

Anemic children received one of the following treatments: Iron supplement (IS), iron plus folic acid supplement (IFS), a multiple micronutrients supplement (MMS), a micronutrient fortified complementary food as porridge powder (FCF), or water fortified with zinc, iron and ascorbic acid (FW). Treatment IS is a standard ferrous sulfate supplement made in liquid solution; IFS is a liquid solution that follows the daily iron and folic acid recommendation of UNICEF to treat anemia [14]; MMS is a supplement with multiple micronutrients specifically designed to treat anemia in low income populations in Mexico [22,23]. The FCF treatment consists of a milk-based complementary food supplement designed for the *Oportunidades *national program (formerly called *Progresa*) in Mexico [16] which currently delivers about five million doses every day. The food supplement is prepared by dissolving the pre-measured powder in 25 mL of boiled water until forming porridge, and it is eaten with a spoon. Lastly, FW treatment is drinking water fortified with iron, zinc and ascorbic acid developed by Procter and Gamble (Cincinnati, OH); mothers were asked to use this water for drinking and cooking for the child. The nutrient composition and chemical form of each treatment is described in **Table **1.

**Table 1:** Nutrient content of experimental treatments

Nutrient	Daily dose by treatment				
	IS	IFS	MMS	FCF	FW*
Iron † (mg)	20	12.5	10	10	6.7
Zinc (mg)			10	10	5.6
Thiamine (mg)			0.6		
Riboflavin (mg)			0.55	0.8	
Pyridoxine (mg)			0.74		
Vitamin B12 (μg)			0.55	0.7	
Folic Acid (μg)		50	37.5	50	
Vitamin C (mg)			30	40	44.4
Vitamin A (UI)				1349	
Vitamin E (mg)				6	

Iron supplement (IS), iron plus folic acid supplement (IFS), multiple micronutrients supplement (MMS), micronutrient fortified complementary food as porridge powder (FCF), or zinc, iron and ascorbic acid fortified water (FW).

*Intake depended on the amount of water used for cooking in each family in addition to drinking water, thus the value represents the average daily intake in the children studied (0.89 L/d).

† FCF form of iron is reduced iron; all other treatments have ferrous sulfate.

All study personnel and participants were blinded to the assignment of supplement treatments but not to fortified foods (FCF and FW), since it was not possible to include these in the blinding scheme. The IS, IFS and MMS treatments were provided in white plastic bottles coded with different letters. All treatments were delivered once a week by field workers and were administered daily to children at home during 4 months. Mothers or caregivers were responsible for the preparation and administration of the treatments. On the first day of the study, fieldworkers explained how to prepare the treatments, and how and when to give them to the children. The fieldworkers visited the participants twice a week at their homes for the duration of the study, and evaluated treatment administration, morbidity, and treatment adherence and acceptance. The presence of any adverse events was recorded by mothers and reported every time the fieldworker visited the child.

### Anthropometry evaluation

All children were weighed and measured at baseline and after four months of receiving the treatment. Children were weighed with an electronic scale (SECA erecta 844, Hamburg, Germany) with no shoes or sweaters to the nearest 100 g. Height was measured with a stadiometer (SECA 216, Hamburg, Germany) with no shoes. Younger children who could not stand still by themselves were weighed with a pediatric scale (SECA 334, Hamburg, Germany) and their supine length was measured within 1 mm using a rigid measuring mat for infants (SECA 210, Hamburg, Germany). Knee height was measured using a Knemometer to the nearest 1 mm. The personnel who measured the children were trained and standardized using calibrated methods and standardized techniques according to the Official Mexican Norm for children's health care (NOM-031-SSA2-1999). Each child was examined by the same person at baseline and after intervention following the same procedures.

### Biochemical measurements

Hemoglobin concentration was measured with a photometer (HemoCue Blood-Hemoglobin System, Ängelholm, Sweden) which was calibrated each time before being used. According to the region altitude of 1600 m and children's age, anemia was defined when Hb < 11.7 g/dL [24,25].

A blood sample (7 mL) was collected from each anemic child after an overnight fast at baseline and after 4 months of treatment to measure iron, ferritin concentrations, and C reactive protein. Blood samples were collected in mineral-free no-additives Vacutainer tubes (Becton Dickinson, Franklin Lakes, NJ) and transported to the laboratory in frozen gel within two hours. Samples were centrifuged at 1500 rpm for 15 min (Beckman Allegra 21R, Palo Alto CA), plasma was separated and aliquotted into 5 mL Eppendorf tubes previously labeled and stored at -70°C until analysis.

Total iron was determined using an atomic absorption spectrophotometer (Perkin Elmer, Mod. Analyst700). Ferritin was determined by immunoradiometric assay (Coat-A-Count Ferritin IRMA; Diagnostic Products). Both analyses were done in triplicate, and the mean value was used for data analysis. Ten samples that had coefficient of variation > 25% were re-analyzed. Low ferritin was defined as serum ferritin < 7 μg/L, and iron deficiency was defined as total iron < 50 μg/dL [25]. The inflammation marker, C-reactive protein (CRP), was measured with a qualitative method described by Ortega-Heredia& Rodríguez-Sánchez[26] and was used to exclude potential false positive values of ferritin and total iron due to inflammation.

### Morbidity evaluation

Morbidity was recorded by using a questionnaire which evaluated general symptoms of upper and lower respiratory tract infections and gastrointestinal infections in the previous 3 or 4 days. This questionnaire has been validated and used in previous studies [27]. Morbidity data was computed as frequency of diarrhea and respiratory infection episodes during the trial period. A diarrhea episode was defined as starting with at least 3 liquid bowel movements per day, and ending the day before the subject did not experience the symptom for 7 consecutive days. The respiratory infection episode was defined as starting when a subject experienced cough, difficulty breathing, ear pain or fever along with a cold, flu or sore throat and ending the day before the subject did not experience any of the previous symptoms.

### Evaluation of adherence and acceptance of the treatment

Once a week, a questionnaire was administered to the mothers or caregivers to evaluate adherence to treatments and the children's acceptance of the different treatments. During that visit, treatments were weighed at the participants' homes with an electronic scale (Ohaus CS2000, Pine Brook, NJ, USA) to the nearest 1 g, except for the FW treatment, which was counted as whole or 1/4, 1/2 or 3/4 fractions of bottles to record and ensure its consumption. However, since the FW was also used for cooking foods that some times were also eaten by other family members, children's adherence to the FW treatment was not possible to evaluate. For the rest of the experimental groups, treatment adherence was computed as the proportion of assigned treatment that was consumed. An adherence value of 80% was considered adequate. The proportion of children that completed 4 months of treatment was also evaluated, regardless of the adherence to their assigned treatment.

To assess acceptability of the treatments, mothers or caregivers answered the following questions: 1) Does your child like to take the treatment? 2) Has your child had any difficulty taking the treatment? Each question was computed as the number of times that the mother reported her child having any difficulty taking the supplement or disliked the treatment. These two outcome variables were used to assess the children's acceptance of the treatments.

### Data analysis

**Figure 1:**
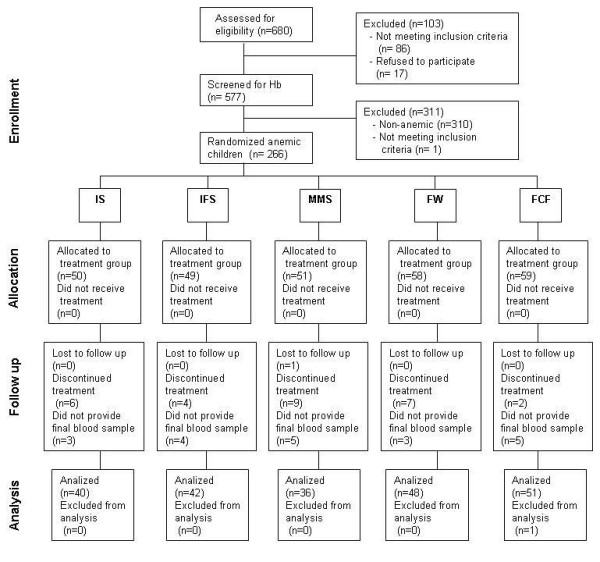
**Flowchart of participants**.

Of the 266 children enrolled in the study, 217 had complete data sets. Statistical analyses were performed with a data set that excluded subjects who did not complete the 17 weeks of study or did not take the treatment (Figure 1). Children (n = 48) that were lost to follow-up did not participate in the final evaluation; thus an intention-to-treat analysis was not possible. Height-for-age, weight-for-age and weight-for-height Z-scores were computed using the WHO child growth standards using the SPSS program provided by WHO [28]. Analyses were performed using SPSS v.10.0 (Chicago, Ill).

To evaluate the effect of treatments on biochemical variables and anthropometry, a univariate analysis of variance was performed with the changes (final - baseline) as the dependent variable, and gender and treatment as fixed effects. Covariates were baseline values, consumed iron (from the treatment formula multiplied by the individual adherence to the treatment) and age; community was included as a random effect. Differences among treatments were tested by using the Least Significant Difference test. Cases with a positive CRP (n = 15) were removed from the data sets for ferritin and total iron.

The effect of treatment on morbidity and child acceptability of the treatment was determined with the generalized linear model procedure with the number of episodes as the dependent variable, assuming the Poisson distribution with the log link function; the model included the variables of age, community, gender and baseline Hb. The Least Significant Difference test was used to compare treatments.

Anemia prevalence at the end of the study, including children that completed the study and that had adherence to the treatment > 80% were compared with an unadjusted chi-square test and with a model assuming the binomial distribution and logit link function adjusted for age and community, gender and baseline Hb.

Analyses were performed in all children and stratified into two groups by gender and by age using a cutoff point of 24 months. Interactions among independent variables were tested and were not significant.

## Results

**Figure 2:**
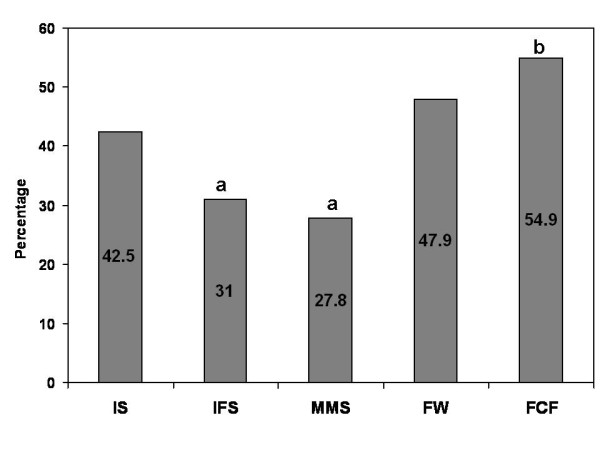
**Post-treatment anemia prevalence of the initially anemic children by treatment**. Treatment abbreviations: Iron supplement (IS), iron plus folic acid supplement (IFS), multiple micronutrients supplement (MMS), micronutrient fortified complementary food as porridge powder (FCF), or zinc, iron and ascorbic acid fortified water (FW) ^a, b ^Different letters represent significant differences among treatments in a Chi Square test (p < 0.05).

A total of 267 children out of the initial 577 sample were anemic. One child had Hb ≤ 7 g/dL (6.0 g/dL), was not included in the study and was referred to the health clinic for further treatment. The flow of participants is shown in **Figure **1. Baseline characteristics of 266 subjects are presented in **Table **2; these characteristics were not significantly different among treatment groups. **Table **3 shows the effect of treatments on Hb, ferritin and total iron. After 4 months, all treatments increased Hb concentration (p < 0.05). Changes in Hb concentrations with treatments MMS, IS and IFS were significantly greater than FCF. In adjusted analysis, only supplements with micronutrients (IFS and MMS) increased Hb more than FCF (p < 0.05). Decrease in prevalence of anemia in each treatment group was as follows: MMS -26/36 (72%); IFS -29/42 (69%); IS -23/40 (58%); FW -25/48 (52%) and FCF -23/51 (45%). A Chi square test showed a lower post-treatment prevalence of anemia in subjects treated with MMS and IFS compared with those treated with FCF (p < 0.05) (**Figure **2). Subgroup analyses in Hb response showed similar treatment effects than the analyses that included all children.

**Table 2:** Characteristics of subjects at baseline in each experimental group

Characteristics	IS	IFS	MMS	FW	FCF
N	40	42	36	48	51
Age (m)	20.3 ± 9.4	20.8 ± 8.9	23.6 ± 9.1	22.4 ± 9.5	21.8 ± 8.9
Boys	42.5	47.6	47.2	52.1	52.9
Ferritin < 7 μg/L	50	31.6	56.3	30.4	44.9
Iron < 50 μg/dL	14.3	11.4	3.3	6.7	16.7
Height for age < -2SD	32.5	19	20	27.1	17.6
Weight for height < -2SD	2.5	7.1	0	4.2	0
Weight for age < -2SD	2.5	0	0	0	0

Values are % or mean ± SD. There were no significant differences among treatments with Chi square or ANOVA.

Treatment abbreviations: Iron supplement (IS), iron plus folic acid supplement (IFS), multiple micronutrients supplement (MMS), micronutrient fortified complementary food as porridge powder (FCF), or zinc, iron and ascorbic acid fortified water (FW).

There are no significant differences among treatment groups with ANOVA or Chi Square tests.

**Table 3:** Treatment effect in hemoglobin, ferritin and total iron concentrations of children

Evaluation	IS	IFS	MMS	FW	FCF	p
**Hemoglobin** **(g/dL)**						
N	40	42	36	48	51	
Baseline	10.45 (10.18, 10.72)	10.72 (10.49, 10.95)	10.73 (10.52, 10.94)	10.55 (10.32, 10.78)	10.51 (10.22, 10.80)	0.487
Final	11.94 (11.60, 12.27)^1^	12.24 (11.90, 12.58)^1^	12.14 (11.80, 12.48)^1^	11.62 (11.28, 11.96)	11.38 (11.07, 11.68)^2^	0.001
Change	1.49 (1.13, 1.84)†^1^	1.52 (1.13, 1.91)†^1^	1.41 (1.05, 1.76)†^1^	1.07 (0.77, 1.36)†	0.86 (0.60, 1.13)†^2^	0.012
Adjusted change *	1.43 (1.05, 1.80)	1.58 (1.26, 1.89)^1^	1.46 (1.08, 1.83)^1^	1.25 (0.89, 1.62)	0.94 (0.65, 1.22)^2^	0.034
**Ferritin** **(μg/dL)**						
N‡	29	32	23	38	46	
Baseline	30.04 (9.90, 50.18)	72.18 (41.64, 102.71)	22.83 (2.97, 42.69)	60.93 (36.55, 85.31)	57.95 (31.18, 84.72)	0.073
Final	55.50 (26.79, 84.22)	51.24 (26.83, 75.64)	20.53 (4.12, 36.94)	48.16 (21.33, 74.98)	60.45 (32.26, 88.63)	0.366
Change	25.47 (3.97, 46.96)†^1^	-20.94 (-44.78, 2.89)^2^	-2.30 (-10.61, 6.01)	-12.77 (-35.49, 9.95)^2^	2.49 (-17.90, 22.89)	0.042
Adjusted change *	21.43 (-4.05, 46.91)	0.94 (-19.50, 21.38)	0.67 (-24.96, 26.30)	-4.06 (-26.80, 18.69)	20.29 (2.97, 37.62)	0.200
**Total Iron** **(μg/dL)**						
N‡	26	30	21	33	46	
Baseline	1.05 (0.90, 1.20)	1.01 (0.86, 1.16)	1.04 (0.88, 1.19)	1.17 (0.99, 1.34)	1.04 (0.90, 1.17)	0.822
Final	1.26 (1.08, 1.45)	1.16 (1.03, 1.29)	1.26 (1.02, 1.50)	1.35 (1.17, 1.53)	1.23 (1.09, 1.38)	0.604
Change	0.21 (0.08, 0.35)†	0.15 (0.04, 0.26)†	0.23 (0.04, 0.42)†	0.19 (0.06, 0.31)†	0.20 (0.10, 0.29)†	0.932
Adjusted change *	0.16 (0.00, 0.31)	0.13 (0.01, 0.25)	0.16 (0.00, 0.32)	0.25 (0.11, 0.39)	0.16 (0.05, 0.26)	0.771

Treatment abbreviations: Iron supplement (IS), iron plus folic acid supplement (IFS), multiple micronutrients supplement (MMS), micronutrient fortified complementary food as porridge powder (FCF), or zinc, iron and ascorbic acid fortified water (FW).

Values are means (95% CI).

* Estimated changes adjusted for initial value, consumed iron in the treatment (according to formula and adherence to the treatment) and community.

† Change is significant within treatment at p < 0.05 in paired T-Test

‡ Excludes children with positive C-Reactive Protein from qualitative analysis

^1,2 ^Different numbers indicate significant difference in the Least Significant Test at p < 0.05.

All treatments were effective in increasing total iron concentration; no significant differences were observed in total iron concentrations among treatments at the end of the study. The IS supplement significantly increased ferritin concentration compared to its baseline value. Unadjusted change in ferritin concentration in the IS treatment group was significantly higher than changes observed in IFS and FW (p < 0.05); however, when adjusting for the amount of consumed Fe and other demographic confounders, the differences were no longer significant.

There were no significant differences among treatment groups in weight-for-age, weight-for-height and height-for-age changes (**Table **4). Similarly, there were no significant effects of treatment on the frequency of gastrointestinal and respiratory infection episodes. The mean diarrhea episodes (95%CI) for IS, IFS, MMS, FW and FCF were: 1.60 (1.21, 1.99), 1.90 (1.45, 2.35), 1.26 (0.87, 1.65), 1.82 (1.42, 2.22) and 1.90 (1.50, 2.31), respectively. The mean respiratory infection episodes (95%CI) for IS, IFS, MMS, FW and FCF were: 1.32 (0.97, 1.67), 1.84 (1.40, 2.27), 1.54 (1.12, 1.96), 1.66 (1.28, 2.03) and 1.69 (1.31, 2.06), respectively.

**Table 4:** Treatment effect in anthropometry measurements of children

Measurement	IS	IFS	MMS	FW	FCF	P
N	40	41	36	48	51	
**Height,** **cm**						
Baseline	78.76 (76.26, 81.27)	79.84 (77.25, 82.44)	82.93 (80.34, 85.52)	80.49 (78.07, 82.91)	80.69 (78.58, 82.79)	0.260
Final	82.31 (80.02, 84.60)	83.63 (81.21, 86.06)	86.44 (84.04, 88.85)	83.97 (81.63, 86.30)	84.37 (82.39, 86.35)	0.219
Change	3.55 (3.06, 4.04)†	3.79 (3.33, 4.25)†	3.51 (3.05, 3.98)†	3.48 (3.11, 3.85)†	3.69 (3.30, 4.07)†	0.822
Adjusted change *	3.55 (3.21, 3.90)	3.67 (3.31, 4.03)	3.68 (3.30, 4.06)	3.71 (3.39, 4.04)	3.70 (3.38, 4.02)	0.971
**Weight,** **Kg**						
Baseline	10.50 (9.81, 11.18)	10.65 (9.97, 11.33)	11.44 (10.75, 12.13)	10.88 (10.24, 11.53)	11.04 (10.49, 11.58)	0.356
Final	11.19 (10.56, 11.81)	11.47 (10.75, 12.20)	12.22 (11.52, 12.92)	11.55 (10.87, 12.23)	11.68 (11.12, 12.25)	0.351
Change	0.69 (0.49, 0.90)†	0.82 (0.63, 1.02)†	0.78 (0.60, 0.96)†	0.67 (0.50, 0.83)†	0.65 (0.47, 0.82)†	0.603
Adjusted change *	0.73 (0.55, 0.90)	0.80 (0.62, 0.98)	0.81 (0.62, 1.00)	0.76 (0.60, 0.93)	0.68 (0.51, 0.84)	0.801
**Knee height,** **cm**						
Baseline	20.52 (19.65, 21.38)	20.65 (19.75, 21.55)	21.76 (20.85, 22.67)	21.03 (20.21, 21.84)	21.12 (20.41, 21.84)	0.332
Final	21.71 (20.89, 22.53)	22.10 (21.28, 22.93)	23.12 (22.25, 23.99)	22.21 (21.42, 22.99)	22.45 (21.76, 23.13)	0.213
Change	1.19 (0.99, 1.39)†	1.45 (1.26, 1.64)†	1.36 (1.12, 1.60)†	1.18 (1.01, 1.36)†	1.32 (1.13, 1.51)†	0.243
Adjusted change *	1.17 (0.99, 1.34)	1.30 (1.12, 1.49)	1.31 (1.12, 1.51)	1.11 (0.94, 1.27)	1.18 (1.02, 1.34)	0.383
**Weight for age,** **Z score**						
Baseline	-0.41 (-0.76, -0.06)	-0.40 (-0.70, -0.10)	-0.32 (-0.63, -0.01)	-0.42 (-0.74, -0.11)	-0.24 (-0.50, 0.02)	0.898
Final	-0.46 (-0.74, -0.17)	-0.39 (-0.68, -0.09)	-0.34 (-0.66, -0.02)	-0.51 (-0.84, -0.19)	-0.36 (-0.62, -0.10)	0.749
Change	-0.05 (-0.23, 0.13)	0.01 (-0.14, 0.17)	-0.02 (-0.11, 0.08)	-0.09 (-0.21, 0.04)	-0.12 (-0.25, 0.01)	0.669
Adjusted change *	-0.02 (-0.15, 0.11)	-0.01 (-0.14, 0.12)	0.00 (-0.14, 0.14)	-0.02 (-0.14, 0.10)	-0.08 (-0.20, 0.04)	0.908
**Weight for height,** **Z score**						
Baseline	0.33 (-0.01, 0.68)	0.28 (-0.01, 0.58)	0.35 (0.05, 0.65)	0.35 (0.04, 0.65)	0.50 (0.25, 0.76)	0.865
Final	0.84 (0.53, 1.15)	0.75 (0.44, 1.07)	0.91 (0.55, 1.27)	0.80 (0.46, 1.14)	0.76 (0.45, 1.08)	0.970
Change	0.51 (0.21, 0.80)†	0.47 (0.19, 0.74)†	0.56 (0.36, 0.75)†	0.45 (0.24, 0.67)†	0.26 (0.03, 0.49)†	0.446
Adjusted change *	0.53 (0.29, 0.76)	0.39 (0.14, 0.64)	0.53 (0.27, 0.78)	0.50 (0.28, 0.72)	0.31 (0.09, 0.53)	0.594
**Height for age,** **Z score**						
Baseline	-1.27 (-1.63, -0.91)	-1.16 (-1.46, -0.86)	-1.09 (-1.41, -0.78)	-1.32 (-1.63, -1.01)	-1.19 (-1.44, -0.94)	0.868
Final	-2.10 (-2.44, -1.76)	-1.83 (-2.24, -1.42)	-1.82 (-2.22, -1.43)	-2.11 (-2.46, -1.76)	-1.80 (-2.13, -1.48)	0.425
Change	-0.83 (-1.11, -0.55)†	-0.67 (-0.96, -0.37)†	-0.73 (-0.94, -0.52)†	-0.79 (-1.01, -0.57)†	-0.61 (-0.86, -0.36)†	0.717
Adjusted change *	-0.81 (-1.06, -0.56)	-0.60 (-0.86, -0.34)	-0.65 (-0.92, -0.37)	-0.74 (-0.98, -0.50)	-0.60 (-0.83, -0.37)	0.710

Treatment abbreviations: Iron supplement (IS), iron plus folic acid supplement (IFS), multiple micronutrients supplement (MMS), micronutrient fortified complementary food as porridge powder (FCF), or zinc, iron and ascorbic acid fortified water (FW). Values are means (95% CI).

* Estimated changes adjusted for initial value and community. Weight, height and knee height are also adjusted for age.

† Change is significant within treatment at p < 0.05 in paired T-Test

No differences were observed among groups in the proportion of children that had an adherence > 80% or in the children that completed the study (**Table **5). The MMS treatment had the lowest acceptability, followed by, IFS and FCF; FW treatment had the fewest reported intake difficulties (p < 0.05).

**Table 5:** Treatment acceptance and adherence

Outcome variables	IS	IFS	MMS	FW	FCF	P
**Acceptance of the treatment:**						
**Trouble taking the treatment **†	3.7 (3.1, 4.2) ^2^	1.9 (1.5, 2.4) ^3^	6.4 (5.5, 7.2) ^1^	1.3 (1.0, 1.6) ^4^	2.3 (1.9, 2.8) ^3^	< 0.001
**Child dislike of the treatment **†	5.1 (4.4, 5.8) ^2^	3.1 (2.6, 3.7) ^3^	7.4 (6.5, 8.4) ^1^	2.0 (1.6, 2.3) ^4^	3.1 (2.6, 3.6) ^3^	< 0.001
**Adherence to the treatment:**						
**Completed 80% of treatment dose, %**	91.5 (83.4, 99.7)	89.2 (79.1, 99.4)	84.6 (71.9, 97.2)	*	89.1 (80.1, 98.0)	0.795
**Completed 4 m of treatment, %**	83.2 (72.8, 93.6)	84.9 (74.5, 95.2)	71.0 (57.7, 84.4)	86.3 (77.1, 95.5)	88.5 (80.0, 97.0)	0.168

Treatment abbreviations: Iron supplement (IS), iron plus folic acid supplement (IFS), multiple micronutrients supplement (MMS), micronutrient fortified complementary food as porridge powder (FCF), or zinc, iron and ascorbic acid fortified water (FW)

Values are estimated means (95%C.I.) adjusted for age and baseline Hb. P value for the treatment main effect evaluated in a generalized linear model assuming the poisson distribution and log link function for adherence and rejection to the treatment variables and the binomial distribution and logit link function for the completion of the study variable. Models were adjusted for community, baseline Hb, age and gender.

* FW treatment does not have an adherence reference parameter, thus this treatment was not included in the model.

† Number of weekly visits that the mother reported difficulty to give the treatment to the child

^1-4^Different numbers indicate significant difference in the Least Significant Test at p < 0.05.

The proportion of children experiencing any adverse event was: IS (4.3%), MMS (10.9%), FCF (5.4%), FW (7.0%), IFS (4.9%). Most of the adverse effects were related to allergies, infections and viral diseases, such as chicken pox. All adverse events were diagnosed by the physician of the health clinic and none of them was related to the allocated treatment.

## Discussion

### Supplements Vs Fortified foods

In the present study, anemic children who received supplements (MMS, IS, and IFS) to treat anemia, had a greater increase in Hb concentration than those who received a fortified complementary food (FCF). When adjusting for the amount of iron consumed, the change in Hb concentration in only the IS group did not differ from that of the FCF group. These results suggest that other factors are more relevant than the amount of iron given to treat anemia, and that the supplements increase Hb concentrations of anemic children more than fortified complementary foods.

Various studies have evaluated the effect of iron supplements or fortified foods on anemia, but few studies have evaluated both in the same trial. Ahmed et al. compared the effect of iron in powder added to foods, and iron in the form of syrup, in children with IDA and found that both groups increased Hb concentration similarly [29]. Thi Le et al. found that the effect of iron fortified noodles on Hb concentration was about half of the maximum impact of iron supplementation [30]. Similar to other trials [18,30,31], this study found that FW and FCF increased Hb concentration and decreased anemia prevalence, but the effect was greater when supplements were used.

### Iron alone Vs Iron + multivitamins

Although the iron dose used to treat the anemic children is twice as large in the IS supplement than the MMS, no differences were found in the change in Hb concentration among IS, MMS and IFS.

A systematic review of several clinical trials concluded that the addition of multiple micronutrients to iron supplementation marginally improved Hb response compared with iron supplementation alone [32]. In the present study, the inclusion of the micronutrients that are known to be involved in erythropoiesis (MMS), or the inclusion of folic acid (IFS), increased Hb in a similar way to iron alone (IS). However, the MMS and IFS treatments were more effective for reducing the prevalence of anemia in children. The prevalence of anemia decreased more in the MMS (14%) and IFS (11%) groups than in the IS group, suggesting that the children included in the study might have had other micronutrient deficiencies that could be contributing to the presence of anemia.

### Effects on morbidity

Iron supplementation has been found to increase the number of diarrhea episodes. Gera and Sachdev reviewed several trials and found that iron supplementation increases the risk of developing diarrhea by 0.05 more episodes per year, which is considered a low risk [33]. Similar to other studies [34,35], this study did not find a difference in diarrhea episodes among treatments.

There is no evidence that iron supplementation has an adverse effect on respiratory tract infections. It has been reported that the inclusion of micronutrients can decrease the number of acute respiratory infections [36]. In the present study, no differences were found in respiratory tract infections among treatment groups; however it cannot be concluded that iron supplementation alone or with micronutrients does not affect respiratory infections due to the lack of a control group with no treatment.

### Effect on growth

No differences were observed on height-for-age, weight-for-age, weight-for-height or knee height among treatments. These findings agree with those of Sachdev et al. who examined the effect of iron supplementation on growth of children in 25 trials [37]. They also did not find that iron supplementation affects growth. Also, it is likely that the period of observation in this study was not long enough to find any differences in growth among treatments.

### Importance of adherence and child rejection

There were no significant differences in adherence rates among treatments. However, the supplements were not as well accepted as the fortified foods (FCF and FW). Therefore, the supplements increased Hb concentrations and were effective, but they were less accepted by the children. Most cases of rejection of the supplements were attributed to flavor, probably due to the high concentration of minerals in the solutions. Careful attention needs to be provided to the sensory characteristics of supplements during their development to minimize rejection and improve adherence to treatment.

### Limitations of the study and implications

In order to compare the efficacy and adherence to different strategies for treating anemia that have been developed in recent years, this study evaluated treatments with different iron content. Results show that the smaller doses, such as 10 mg per day, are as effective as larger doses to increase Hb and to reduce the prevalence of anemia. Children from 6 to 42 months of age were studied and, since all children were non-breastfed or partially breastfed, our findings cannot be generalized to exclusively breastfed children. In rural communities in Mexico, only about 10% of children are still exclusively breastfed between 9 to 12 months [38]. Another limitation of the present study is that treatments were consumed for 4 months; some of the treatments may have been more effective if they had been consumed for longer periods of time.

## Conclusions

After evaluating the efficacy of different strategies to treat anemia for 4 months it can be concluded that in Mexican children living in rural areas where the prevalence of anemia is high: 1) All treatments evaluated increased Hb and reduced the prevalence of anemia. 2) The three supplements (iron, iron plus folic acid and iron plus micronutrients) increased Hb concentrations significantly more than fortified complementary food. 3) The supplements that contained other micronutrients or folic acid were more effective for reducing the prevalence of anemia than fortified complementary food. 4) Supplements were in general less accepted by children than the food fortification strategies. This study demonstrates that it is more important to consider other micronutrients than to provide high doses of iron alone. We suggest that attention should be given to other micronutrient deficiencies to increase the effectiveness of iron intervention programs.

## List of Abbreviations

IDA: Iron deficiency anemia; IS: Iron alone supplement; IFS: Iron plus folic acid supplement; MMS: Multiple micronutrients supplement; FCF: Micronutrient fortified complementary food as porridge powder; FW: Zinc plus iron plus ascorbic acid fortified water; Hb: Hemoglobin; WHO: World Health Organization; UNICEF: United Nations Children's Fund

## Competing interests

The authors declare that they have no competing interests.

## Authors' contributions

JLR participated in the design of the study, interpretation of results and critically revised the manuscript. KEG controlled the fieldwork and drafted the manuscript. MCP participated in managing data, performed the statistical analyses and contributed to the manuscript. OPG revised the manuscript critically for important intellectual content. RP performed all the biochemical analysis. MO contributed with the conception and design. All authors read and approved the final manuscript.
